# WD repeat domain 76 predicts poor prognosis in lower grade glioma and provides an original target for immunotherapy

**DOI:** 10.1186/s40001-023-01605-6

**Published:** 2024-01-03

**Authors:** Xingbo Cheng, Zhendong Liu, Haigang Chang, Wenjia Liang, Pengxu Li, Yanzheng Gao

**Affiliations:** 1 Department of Surgery of Spine and Spinal Cord, Henan Provincial People’s Hospital, People’s Hospital of Zhengzhou University, People’s Hospital of Henan University, No. 7 Weiwu Road, Jinshui District, Zhengzhou, 450003 Henan China; 2 Department of Neurosurgery, The First Affiliated Hospital of Xinxiang Medical University, Weihui, 453100 Henan China; 3 People’s Hospital of Henan University, Henan Provincial People’s Hospital, Zhengzhou, 450003 Henan China

**Keywords:** *WDR76*, LGG, Prognosis, Cell cycle, Tumor immunity

## Abstract

**Background:**

The WD40 repeat (WDR) domain provides scaffolds for numerous protein–protein interactions in multiple biological processes. WDR domain 76 (*WDR76*) has complex functionality owing to its diversified interactions; however, its mechanism in LGG has not yet been reported.

**Methods:**

Transcriptomic data from public databases were multifariously analyzed to explore the role of *WDR76* in LGG pathology and tumor immunity. Laboratory experiments were conducted to confirm these results.

**Results:**

The results first confirmed that high expression of *WDR76* in LGG was not only positively associated with clinical and molecular features of malignant LGG, but also served as an independent prognostic factor that predicted shorter survival in patients with LGG. Furthermore, high expression of *WDR76* resulted in the upregulation of oncogenes, such as *PRC1* and *NUSAP1*, and the activation of oncogenic mechanisms, such as the cell cycle and Notch signaling pathway. Finally, *WDR76* was shown to be involved in LGG tumor immunity by promoting the infiltration of immune cells, such as M2 macrophages, and the expression of immune checkpoints, such as *PDCD1* (encoding PD-1).

**Conclusions:**

This study shows for the first time the diagnostic and prognostic value of *WDR76* in LGG and provides a novel personalized biomarker for future targeted therapy and immunotherapy. Thus, *WDR76* may significantly improve the prognosis of patients with LGG.

**Supplementary Information:**

The online version contains supplementary material available at 10.1186/s40001-023-01605-6.

## Background

Malignant gliomas are the most common type of brain cancer, and according to the 2016 World Health Organization (WHO) classification, grade II and grade III gliomas with specific molecular features are classified as lower grade gliomas (LGGs), and grade IV glioma (glioblastoma) is often considered as high grade glioma [1]. The deterioration of patients is inextricably linked to the grades of glioma [2]. Because of the complexity of glioblastoma' pathology, designing a comprehensive treatment plan to completely cure the patient is unfortunately out of reach for now, which is the root cause of the unsatisfactory effects of current glioma treatments [3]. However, LGG, an intermediate transitional state of glioma, has a great potential for the development into higher levels of glioma [4]. Researches focusing on the unique heterogeneity of LGG can shed more light on the specific mechanisms underlying the malignant progression of glioma. Therefore, exploring the pathogenesis of LGG in detail can truncate the ascension pathway for glioma malignancy, and the discovery of biomarkers for personalized treatment may remedy the current precarious state of therapy and improve the prognosis of patients with LGG.

The pathogenesis of LGG results from a combination of disorders involving multiple cellular processes, including signal transduction, epigenetic regulation of gene expression, cell growth and division, and DNA damage sensing and repair [5]. Proteins are ultimately responsible for all biological activities in cells, and the protein–protein interactions ensure the cellular functioning of active substances [6]. In the human proteome, WD40 repeat (WDR) domain-containing proteins form one of the largest superfamilies, providing WDR domains as scaffolds for protein–protein interactions, and participating in the pathogenesis of numerous cancers [7]. For example, increased WDR54 expression in colorectal cancer (CRC) leads to shorter disease-specific survival and has been identified as an independent prognostic factor for CRC [8]. In addition, after the knockdown of the high expression of WDR43 in CRC, the ability of cells to proliferate, invade, and migrate is significantly impaired, and tumorigenesis in animal models is reduced [9]. The WDR domain 76 (*WDR76*) protein shares the same WDR domain with other family members, and currently, ongoing research on *WDR76* is gradually enriching the understanding of its functions in differential pathophysiology; however, there have been no reports in the field of LGG.

This study aimed to report the prognostic value of *WDR76* in LGG and its involvement in tumor immunity. Analysis of large amounts of LGG sequencing data from authoritative databases revealed that abnormally high expression of *WDR76*, which is regulated by DNA methylation, is positively associated with malignant features of LGG. Moreover, *WDR76* can be a novel independent risk factor for LGG, and it can also lead to the malignant progression of LGG through its involvement in multiple cancer pathways. Finally, *WDR76* is found to be involved in the immune microenvironment by regulating immune cell infiltration. This study further expands the molecular function of *WDR76* in LGG pathogenesis and tumor immunity, revealing the underlying mechanisms of the malignant progression of LGG and opening up a wider field for future research. As the pathological networks underlying LGG heterogeneity are revealed, it will revolutionize therapies for LGG and lead to a satisfactory outcome for patients.

## Materials and methods

### Data collection using public databases

This study analyzed the potential diagnostic and prognostic value of *WDR76* by collecting transcriptomic data of thousands of patients with LGG, using multiple authoritative databases. First, the Gene Expression Profiling Interactive Analysis (GEPIA) database was used to explore differences in *WDR76* expression between cancerous and normal tissues. Second, the Human Protein Atlas (HPA) database was used to verify the protein levels of *WDR76* in different tissues. Third, LGG sequencing data of 503 cases from The Cancer Genome Atlas (TCGA) and 403 cases from The Chinese Glioma Genome Atlas (CGGA) with complete clinical information were collected to uncover the relationship between *WDR76* expression, clinical characteristics, and patient prognosis (Additional file 1: Tables S1, S2). LGG methylation data from TCGA, detected using the Illumina HumanMethylation450 BeadChip, were used to validate the above analysis. Each probe ID corresponded to a CpG site, followed by beta values representing methylation levels ranging from 0 to 1, with 0 representing complete demethylation and 1 representing complete methylation. In addition, for meta-analysis, we also collected LGG data from the GSE4412 (26 cases) and GSE43378 (18 cases) data sets composing from the Gene Expression Omnibus database. Finally, the Tumor Immune Estimation Resource (TIMER) database was used to determine the influence of *WDR76* in the immune microenvironment.

### Co-expression analysis and gene set enrichment analysis

To elucidate the detailed mechanism of *WDR76* involvement in LGG pathological progression at the molecular level, co-expression analysis was performed to investigate the correlation between *WDR76* expression and other genes. In addition to the induced alterations of gene expression, the biological function of *WDR76* in LGG with respect to signaling pathways was also identified using Gene Set Enrichment Analysis (GSEA). All LGG data were categorized into high- and low-expression groups according to the *WDR76* expression median, and gene-set enrichments were analyzed in both groups using GSEA 4.0 jar software. Based on *p* < 0.05, and false discovery rate < 0.25, enrichment results in a highly expressed *WDR76* phenotype were considered the signaling pathway activated by *WDR76* in LGG.

### Meta-analysis

To systematically study the effects of *WDR76* on LGG prognosis, we attempted to integrate all relevant studies on *WDR76*. However, by searching multiple databases, such as the Web of Science and the National Center for Biotechnology Information, we found no reports of *WDR76* in LGG. Therefore, we combined LGG data from TCGA (503 cases), CGGA (403 cases), GSE4412 (26 cases), and GSE43378 (18 cases) data sets for the meta-analysis. The consistency of the data from multiple databases was explored to confirm the impact of *WDR76* on the prognosis of patients with LGG. The heterogeneity of these data sets was evaluated using *Q* assays (*I*^2^ statistics) and a random-effects model was established for the high heterogeneity of the data sets (*I*^2^ > 50%, *p* < 0.05).

### Cell culture and treatment

The LGG cell line SHG-44, purchased from BLUEFBIO, was used to verify whether *WDR76* expression was regulated by DNA methylation. First, SHG-44 cells were cultured in a cell dish with a complete culture medium containing 10% fetal bovine serum and 1% penicillin and streptomycin, and incubated at 37 ℃ in an incubator containing 5% CO_2_. Cell passage was performed when the cell fusion was approximately 80% and the complete medium was replaced as required.

### Real-time quantitative polymerase chain reaction

Real-time quantitative polymerase chain reaction (RT-qPCR) was performed to validate the changes in the mRNA levels of *WDR76* in four normal brain tissues and six LGG samples. Total RNA extracted from tissue samples was synthesized into cDNA via reverse transcription, and then subjected to RT-qPCR assay. The specific primer sequences used in the experiment were as follows: For *WDR76*, forward 5′-TGGGATTGGATGTAGAAGGT-3′, reverse 5′-CTACTAATGTCGGCGGTGTT-3′; for 18S, an internal reference, 5′-GTAACCCGTTGAACCCCATT-3′, reverse 5′-CCATCCAATCGGTAGTAGCG-3′. GraphPad Prism 9 software was used for the statistical analysis and mapping of the processed cycle threshold values of the PCR assay. The experiments involving samples were approved by the Ethics Committee of Henan Provincial People’s Hospital (2020107).

### Statistical analysis

In the present study, Chi-square tests were used to analyze the relationship between *WDR76* expression, methylation data, and the clinical characteristics of LGG. Second, Kaplan–Meier (KM) survival curves were used to reveal the influence of *WDR76* expression on the overall survival (OS) of patients with LGG, and receiver operating characteristic (ROC) curves were drawn to show the diagnostic value of *WDR76* based on 1 year, 3 year, and 5 year survival rates. Third, Cox regression analysis was performed to determine whether *WDR76* is an independent prognostic factor for LGG. Finally, the Estimation of STromal and Immune cells in MAlignant Tumour tissues using Expression data (ESTIMATE) was used to analyze the relationship between *WDR76* expression and the abundance of infiltration of numerous immune cells, and Pearson’s correlation coefficient was used to reveal the correlation of *WDR76* expression with biomarkers of immune cells and with immune checkpoints. All raw data collected from public databases were analyzed using R software (v4.1.0), and *p* < 0.05 was considered statistically significant.

## Results

### Aberrant expression of *WDR76* in LGG

**Fig. 1:**
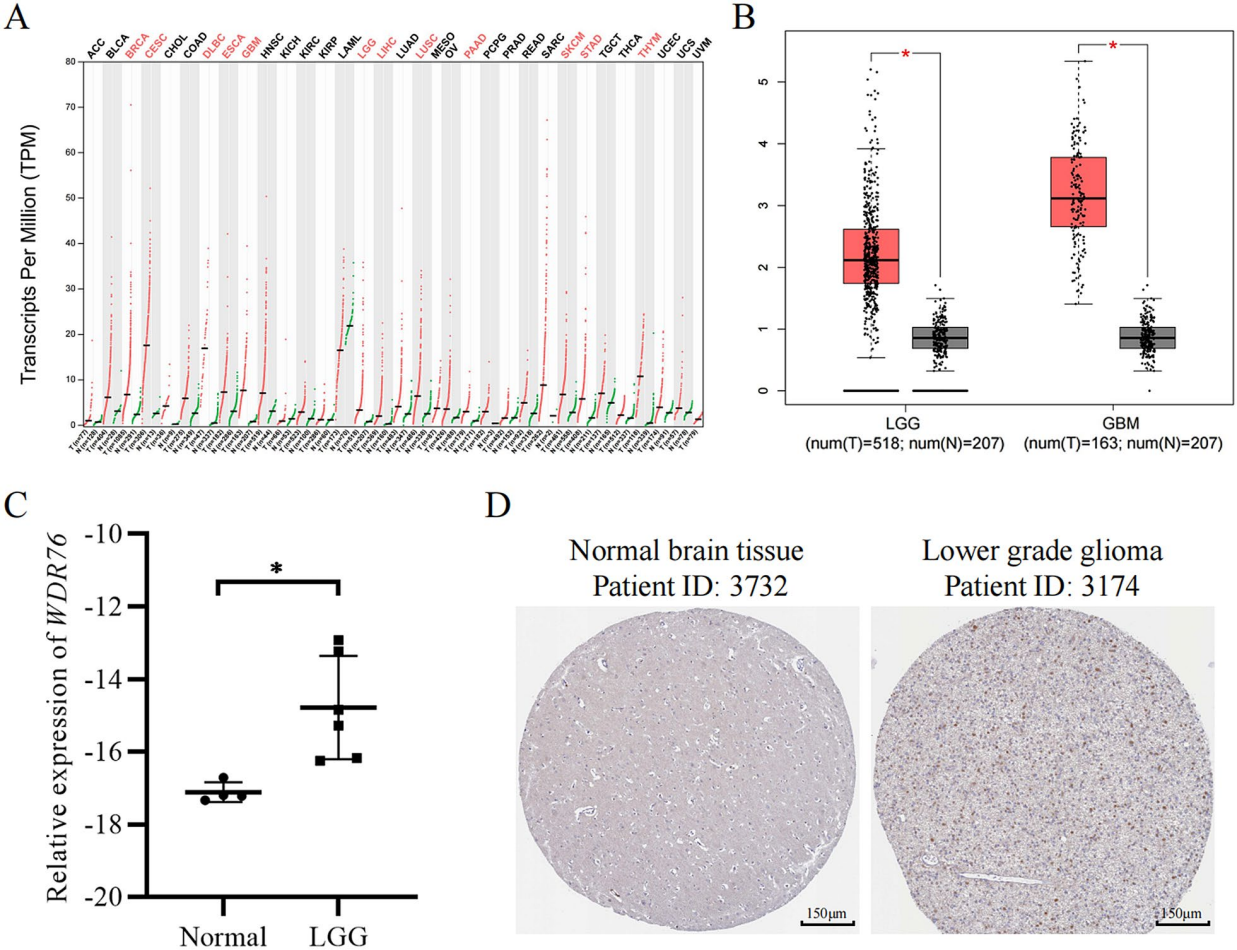
Aberrant expression of *WDR76* in LGG. **A**, **B** Expression levels of *WDR76* in multiple cancers. High expressions are marked red. **C** mRNA levels of *WDR76* in LGG samples (6 cases) and normal brain tissues (4 cases). **D** Protein levels of *WDR76* in the LGG sample and normal brain tissue. * *p* < 0.05

Abnormal gene expression is a hallmark of cancer progression, and the abnormally high expression of pathogenic genes is a major driver of the malignant progression of LGG. The purpose of this study was to elucidate the driving role of *WDR76* in LGG pathogenesis; therefore, we first validated *WDR76* expression levels in LGG. The results of the GEPIA database showed widespread and significantly high expression of *WDR76* in numerous malignant cancers (Fig. 1A), including the LGG (Fig. 1B). These results suggest that a consistent pattern of *WDR76* expression may contribute to the broad pathogenesis of multiple cancers. To validate the database analysis, LGG samples from the laboratory were used to detect the mRNA level of *WDR76*. PCR results showed that *WDR76* expression was significantly higher in the six LGG samples than in the four normal brain tissues (Fig. 1C). Furthermore, immunohistochemistry results obtained from the HPA database showed a significant increase in *WDR76*-stained positive cells compared with normal brain tissues (Fig. 1D). These findings reinforce the irrefutable results of abnormally high expression of *WDR76* in LGG and provide a solid basis for further research.

### High expression of *WDR76* predicts clinical characteristics of malignant LGG

**Fig. 2:**
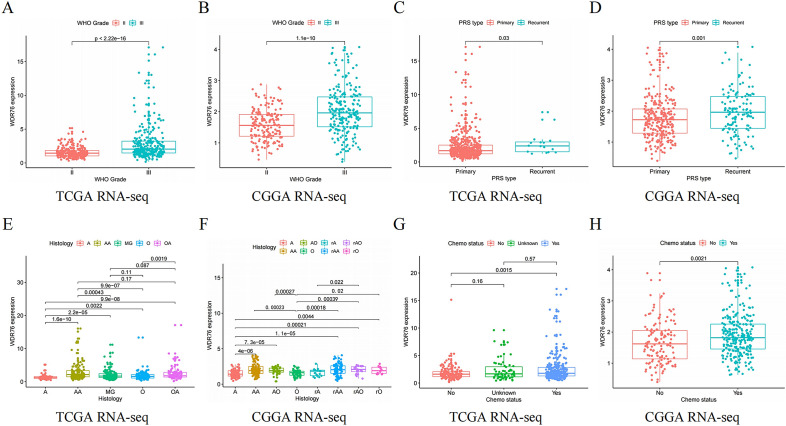
Correlation between *WDR76* expression and LGG clinical features based on TCGA database and CGGA database. **A**, **B** WHO Grade. **C**, **D**, PRS type. **E**, **F** Histology. **G**, **H** Chemo status. *WHO* World Health Organization, *PRS* primary–recurrent–secondary, *chemo* chemotherapy

Highly expressed pathogenic genes drive the deleterious progression of LGG, which is clinically characterized by a higher grade, a more malignant histological type, and tumors that are more prone to recurrence. In this study, consistent results from both TCGA and CGGA databases demonstrated that higher expression of *WDR76* was found in Grade III LGG than in Grade II LGG (Fig. 2A, B), in addition to significantly higher expression of *WDR76* in recurrent LGG than in primary tumors (Fig. 2C, D). Moreover, the highest expression of *WDR76* was found in the histological type of anaplastic astrocytoma (AA) in TCGA database and in recurrent anaplastic oligodendroglioma (rAO) in the CGGA database (Fig. 2E, F). Furthermore, analysis of both databases also showed that high expression of *WDR76* was concentrated in patients receiving chemotherapy (Fig. 2G, H), older patients with LGG and patients receiving radiotherapy (as per the TCGA database) (Additional file 1: Fig S1A, B). In summary, the association between these clinical features demonstrates the pivotal role of *WDR76* in increasing the malignancy of LGG, inevitably leading to poor patient outcomes.

### High expression of *WDR76* is an independent prognostic indicator for LGG

**Fig. 3:**
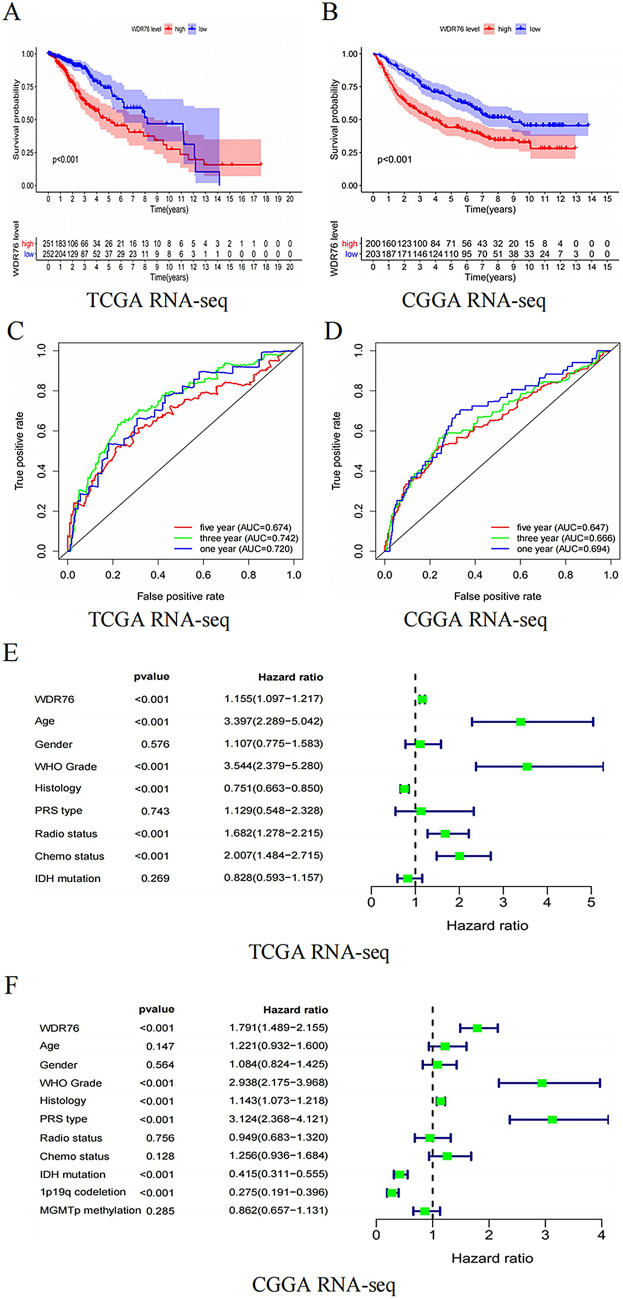

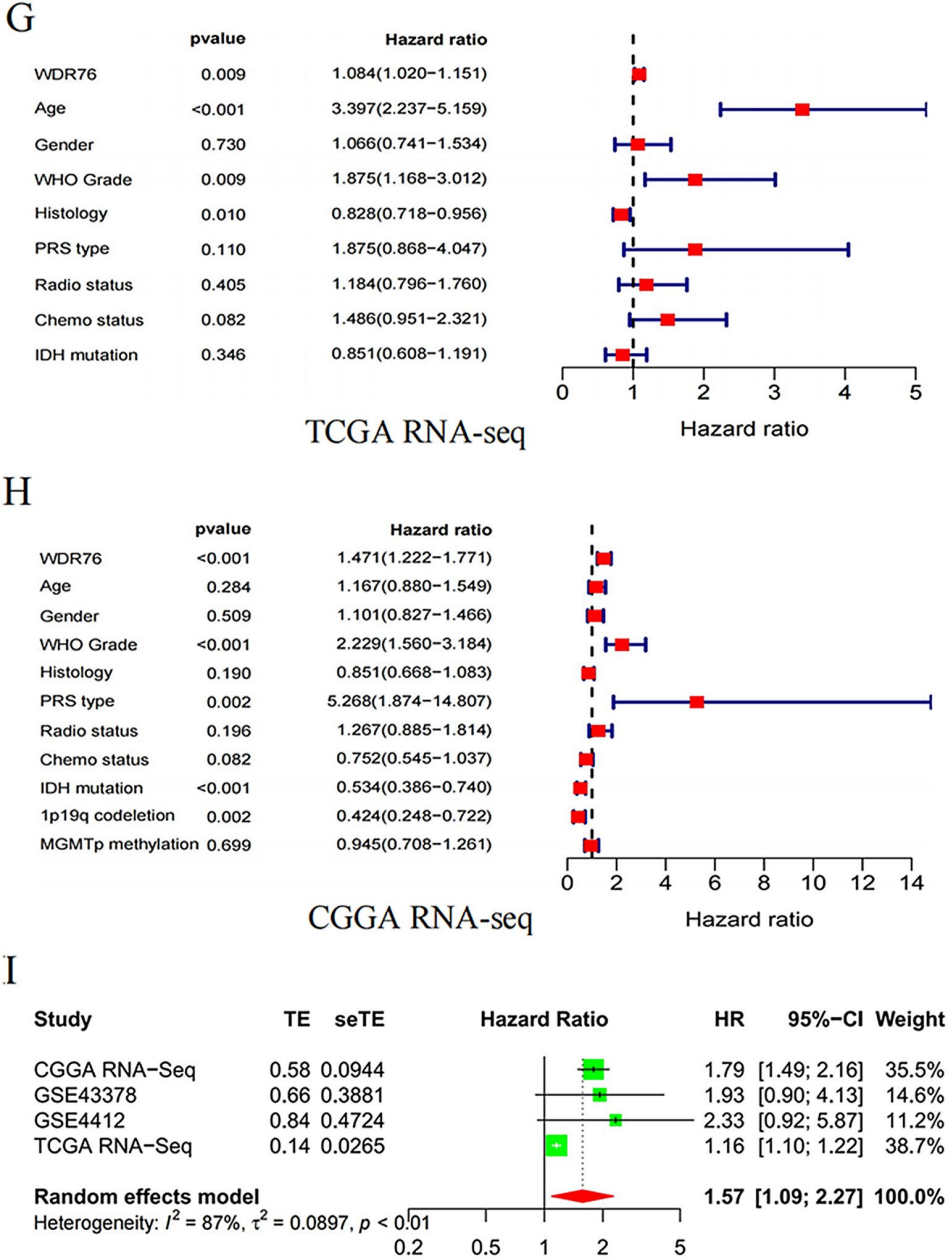
Diagnostic and prognostic value of *WDR76* for LGG based on TCGA and CGGA database. **A**, **B** Overall survival of LGG patients with high and low expressions of *WDR76*. **C**, **D** ROC curves of 1 year, 3 year and 5 year survival rate for LGG patients with *WDR76* expression. **E**, **F** Results of univariate analysis on multiple features for the prognosis in LGG patients. **G**, **H** Results of multivariate analysis on multiple features for the prognosis in LGG patients. I Meta-analysis on *WDR76* for the prognosis in LGG patients based on TCGA database, CGGA database, GSE43378 data set, and GSE4412 data set

Pathogenic genes promote an increase in cancer malignancy, aggravate the patient’s condition, and lead to poor treatment effects, ultimately shortening patient survival. Because the above results revealed a relationship between *WDR76* and LGG malignancy, we further explored the effect of *WDR76* on the prognosis of patients with LGG. First, the KM method showed that patients with LGG with higher expression of *WDR76* had shorter OS in both the TCGA and CGGA databases (Fig. 3A, B). Further analysis of LGG subtypes indicated that the consistent effect of *WDR76* expression on survival remained in Grade III LGG (Additional file 1: Fig S1C, E), while the effect was not statistically significant in patients with Grade II LGG (Additional file 1: Fig S1D, F). However, the results containing accurate molecular subtypes showed that high expression of *WDR76* could lead to shorter survival for patients with LGG with wild-type isocitrate dehydrogenase (IDH), IDH mutation without 1p19q co-deletion, and IDH mutation with 1p19q co-deletion in the CGGA database (Additional file 1: Fig S1G–I). Second, ROC curves confirmed that *WDR76* expression was significantly associated with the 1 year, 3 year, and 5 year survival rate of patients with LGG. Besides, similar results were obtained for LGG with different grades and diverse molecular characteristics (Additional file 1: Fig S1J–P).

Furthermore, in a transverse study, Cox regression analysis was used to screen for factors that affecting the prognosis of patients with LGG. Based on the results of univariate analysis (Fig. 3E, F), multivariate analysis showed that *WDR76* and WHO grade were independent prognostic risk factors for patients with LGG after excluding other confounding factors in both the TCGA and CGGA databases, whereas IDH mutation and 1p19q co-deletion were recognized as protective factors (Fig. 3G, H). Finally, the study selected *WDR76* for the analysis to validate its prognostic impact in patients with LGG. A meta-analysis based on four separate data sets (TCGA, 503 cases; CGGA, 403 cases; GSE4412, 26 cases; and GSE43378, 18 cases) reinforced the status of *WDR76* as a risk factor for prognosis in patients with LGG (Fig. 3I). Taken together, the abnormally high expression of *WDR76* was identified as an inevitable contributor to the poor prognosis in LGG.

### *WDR76* methylation negatively regulates its expression and predicts better prognosis in patients with LGG

**Fig. 4:**
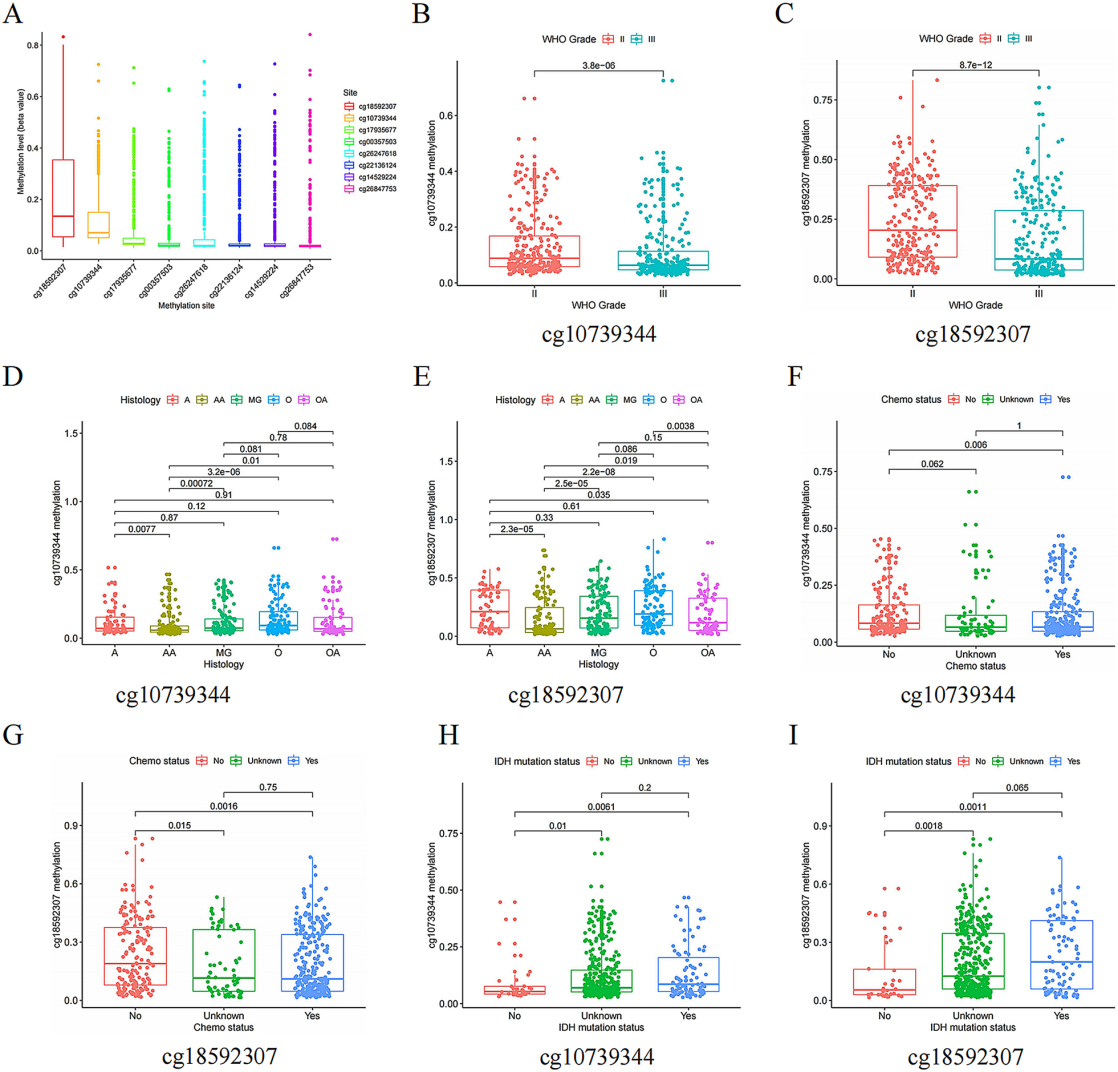

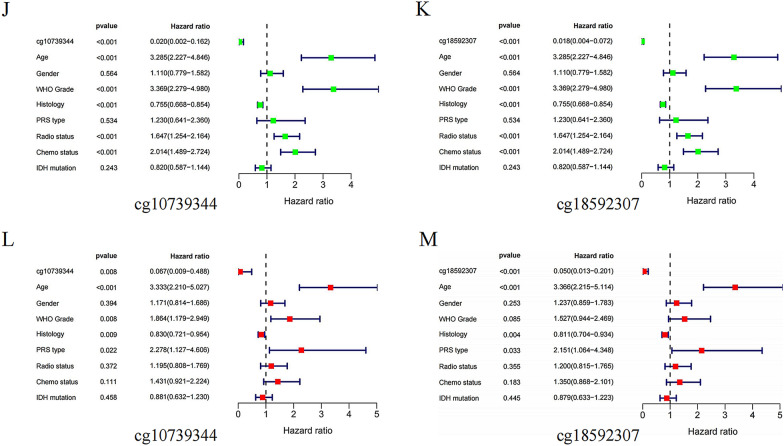
Correlation between cg10739344 and cg18592307 expression with clinical characters and overall survival of LGG patients. **A** Low methylation levels of 8 CpG cites in *WDR76*. **B**, **C** WHO Grade. **D**, **E** Histology. **F**, **G** Chemo status. **H**, **I** IDH mutation status. **J**, **K** Results of univariate analysis on multiple features for the prognosis in LGG patients. **L**, **M** Results of multivariate analysis on multiple features for the prognosis in LGG patients. *WHO* World Health Organization, *Chemo* chemotherapy, *IDH* isocitrate dehydrogenase

With the development of cancer epigenetic in cancer research, the role of DNA methylation in regulating the expression of key cancer-related genes has become increasingly important. Eight methylation sites, all with hypomethylation levels, were identified in the study of the regulatory mechanisms underlying the abnormally high expression of *WDR76* in LGG (Fig. 4A). Subsequent correlation analysis of clinical characteristics found that the hypomethylation levels of two methylation sites, cg10739344 and cg18592307, were significantly associated with malignant clinical features, such as WHO Grade III, histological type AA (Fig. 4B–G), and malignant molecular signature, wild-type IDH (Fig. 4H, I). Similar results were obtained for cg14529224 and cg17935677 (Additional file 1: Fig S2A–F). Moreover, hypermethylation of cg10739344 and cg18592307 sites resulted in longer survival of patients with LGG (Additional file 1: Fig S2G, H), and both sites served as independent protective factors for LGG (Fig. 4J–M). In addition, other methylation sites, such as cg14529224, cg17935677, cg22136124, and cg26247618, also play the same regulatory role in LGG prognosis (Additional file 1: Fig S2I–L).

### Co-expression analysis of *WDR76* and enrichment results of signaling pathways

**Fig. 5:**
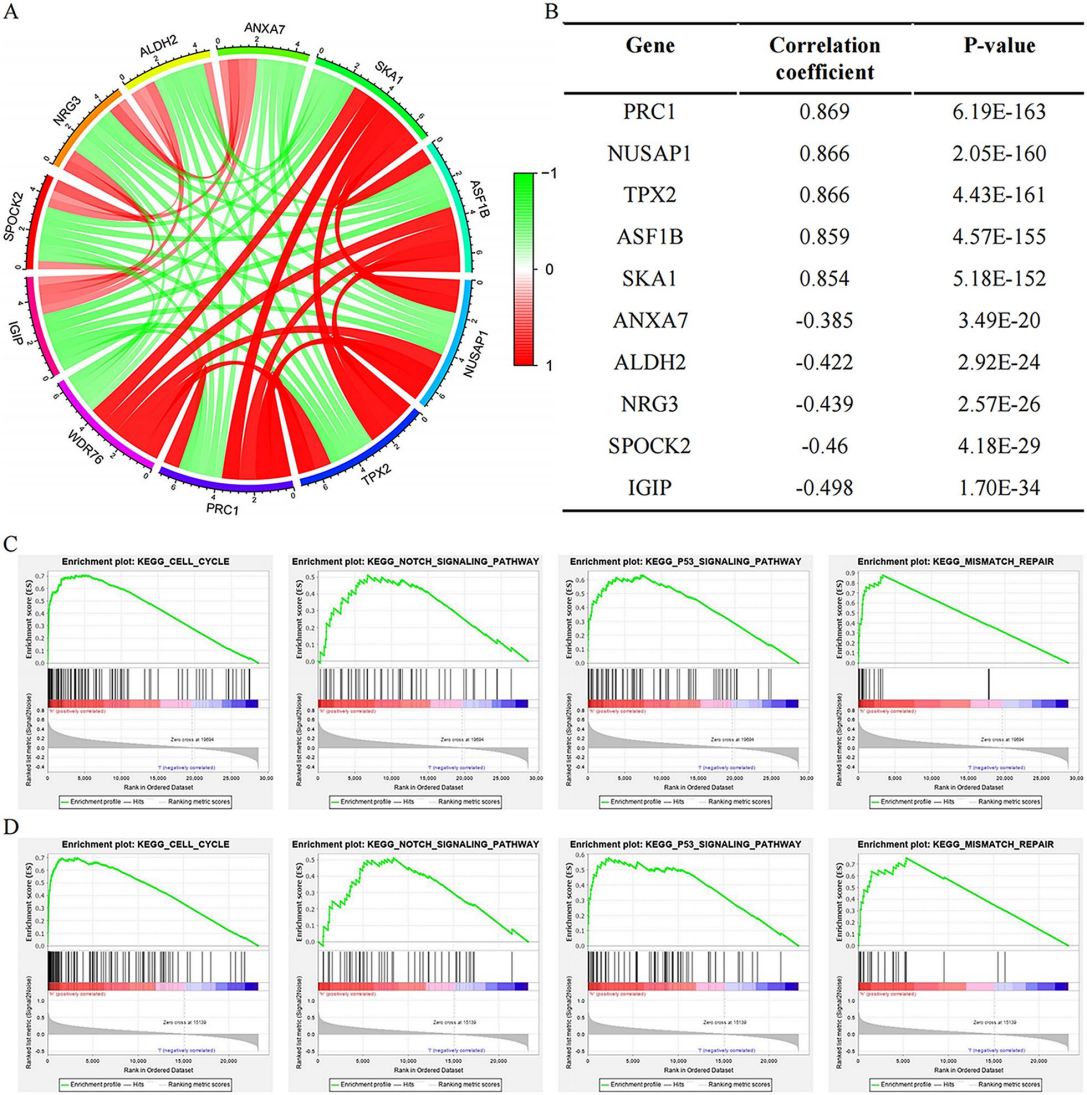
Results of co-expression analysis and GSEA analysis on *WDR76*. **A**, **B** Most related co-expressed genes of *WDR76*. **C**, **D** Pathway enrichment of *WDR76* based on TCGA database and CGGA database

After uncovering the regulatory mechanisms underlying the high expression of *WDR76*, we aimed to elucidate the mechanisms by which *WDR76* high expression is involved in the progression of LGG malignancy. Genomic disorders are at the root of poor LGG progression. Therefore, this study first analyzed the molecular alterations induced by *WDR76*. Co-expression analysis identified five genes (*PRC1*, *NUSAP1*, *TPX2*, *ASF1B*, *SKA1*) with the most positive correlation and five genes (*ANXA7*, *ALDH2*, *NRG3*, *SPOCK2*, *IGIP*) with the most negative correlation with *WDR76* (Fig. 5A, B). Genes with the same expression patterns may activate the same or similar downstream pathways that contribute to LGG progression. Next, we demonstrated the enrichment of signaling pathways with a phenotype of high *WDR76* expression using GSEA. Based on the TCGA and CGGA databases, potential pathways in which *WDR76* may participate included the cell cycle, Notch signaling pathway, p53 signaling pathway, and mismatch repair (Fig. 5C, D) (Additional file 1: Table S3). These pathways, which have been extensively studied in cancer progression, demonstrate strong oncogenic potential, and their role in cancer immunity is being continuously investigated.

### *WDR76* promotes immune infiltration and positively correlates with immune markers

**Fig. 6:**
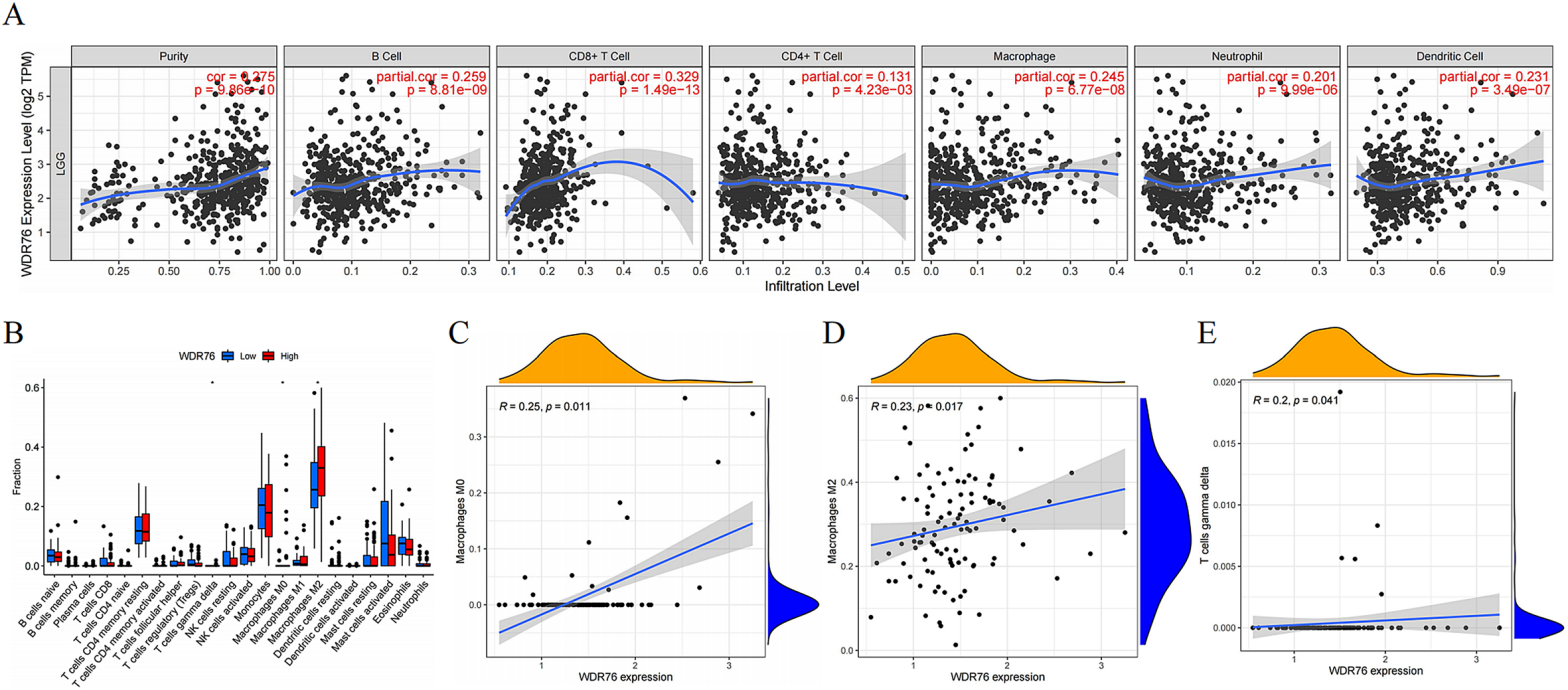
Correlation between *WDR76* expression and immune cells infiltration in LGG. **A** TIMER database indicates the relationship of *WDR76* expression and the infiltration of six immune cells (B cell, CD8 + T cell, CD4 + T cell, macrophage, neutrophil, and dendritic cell). **B** Results of ESTIMATE analysis on the infiltration of different subtypes of immune cells with high and low *WDR76* expressions. **C** Macrophage M0. **D** Macrophage M2. **E** T-cell gamma–delta

**Fig. 7:**
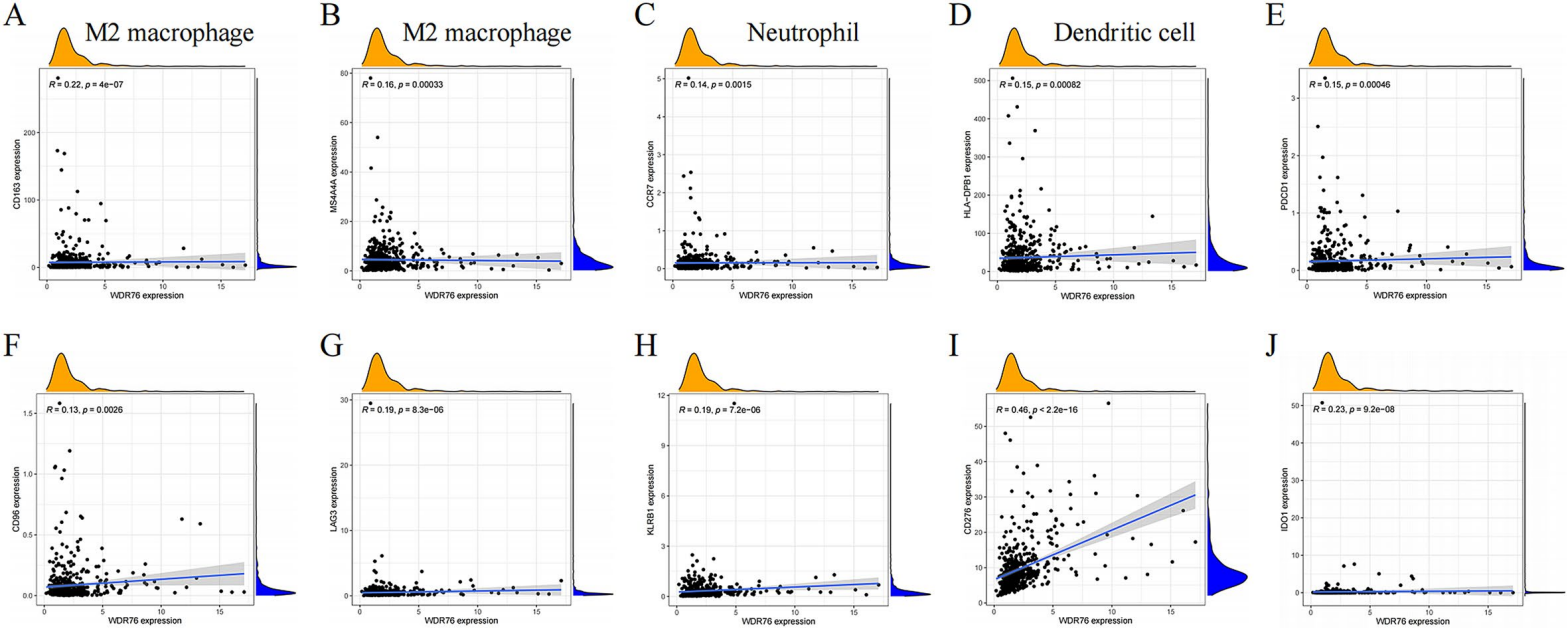
Correlation between the expression of *WDR76* with markers of immune cell subtypes and immune checkpoints. **A** *CD163*. **B** *MS4A4A*. **C** *CCR7*. **D** *HLA*–*DPB1*. **E** *PDCD1*. **F** *CD96*. **G** *LAG3*. **H** *KLRB1*. **I** *CD276*. **J** *IDO1*

With the clinical application of immunotherapy, the paradigm of cancer treatment has been rewritten owing to advances in cancer immunology. As important cellular components of the immune microenvironment, immune cells play an essential character in the regulation of tumor immunity. Therefore, this study explored the relationship between *WDR76* expression and the infiltration of six immune cells using the TIMER database and confirmed that high expression of *WDR76* increased the infiltration levels of immune cells (B cell, CD8 + T cell, CD4 + T cell, neutrophil, macrophage and dendritic cell) (Fig. 6A). In addition, immune cell infiltration promoted by *WDR76* expression also predicted a poor prognosis in patients with LGG (Additional file 1: Fig S3). Moreover, since different subtypes of immune cells perform distinct functions in the tumor microenvironment, we further explored the relationship between *WDR76* expression and the infiltration of specific tumor cell subtypes using the ESTIMATE analysis (Fig. 6B–E). These results demonstrated that *WDR76* expression promoted the infiltration of immune cell subtypes that significantly regulated the immune microenvironment, such as M0 macrophages, and promoted their polarization to M2. Subsequent correlation analysis confirmed a significant positive correlation between the M2 macrophage markers (*CD163* and *MS4A4A*) and *WDR76* (Fig. 7A, B). In addition to its positive relationship with neutrophil and dendritic cell markers (Fig. 7C, D), *WDR76* was showed the same expression trend as many well-known immune checkpoint proteins, including *PDCD1* (encoding PD-1) (Fig. 7E–J). Taken together, this study revealed an irreplaceable role of *WDR76* in regulating tumor immunity in LGG, which may lead to new opportunities for immunotherapy in patients.

## Discussion

As one of the richest domains in the human genome, the WDR domains, with low sequence conservation and functional diversity, are involved in many cellular biological processes, including carcinogenesis [7]. With the increase in cancer-related research, the function of *WDR76*, which contains a WDR domain at the protein C-terminus has been demonstrated [10]. As a cancer suppressor in hepatocellular carcinoma (HCC), *WDR76* acts as an E3 ubiquitin ligase and mediates the polyubiquitination of RAS, leading to the inhibition of proliferation, transformation, and invasion of liver cancer cells [11]. Similar results have also confirmed that *WDR76* causes instability of the RAS in CRC, impairing the proliferation and activation of cancer stem cells by inhibiting the Wnt/β-catenin signaling pathway [12]. Nevertheless, an opposing argument claims that *WDR76* specifically acts as an oncogenic factor in lung adenocarcinoma, which is an independent risk factor for prognosis, and that high expression of *WDR76* is significantly associated with immune cell infiltration, thereby participating in tumor immunity [13]. These controversial results illustrate the complexity of *WDR76* function in different cancer types. The present study is the first to reveal the definitive role of *WDR76* in LGG carcinogenesis and tumor immunity.

To determine the positive and negative properties of *WDR76* in LGG, we first investigated the relationship between *WDR76* expression and clinical characteristics of LGG. The results showed that the high expression of *WDR76* was associated with more aggressive features of LGG, such as a higher WHO grade, malignant histological characteristics, and recurrent LGG. It is well-known that all these malignant features have significant negative effects on patient survival [14], and the high expression of *WDR76* shortened the OS of patients with LGG. Taken together, *WDR76* should be considered an undisputed malignancy factor responsible for poor patient outcomes in LGG. *WDR76* expression was significantly elevated in patients who required chemotherapy. This is in part, because *WDR76* leads to a malignant development in LGG, which cannot be treated solely by surgery but requires adjuvant chemotherapy. However, is that *WDR76* has been reported to preserve genomic stability by being rapidly recruited to DNA damage sites and binding to key proteins involved in DNA damage repair [15–17], which is a mechanism that antagonizes the effect of chemotherapy drugs on DNA damage in LGG cells. We identified the role of WDR family genes in LGG and found that *WDR76* was significantly associated with LGG prognosis and diagnosis, suggesting reliance on the WDR domain and that *WDR76* may have a unique role in participating in LGG pathogenesis. Therefore, *WDR76* can be a promising therapeutic target to complement radiotherapy and improving the prognosis of patients with LGG.

Survival time is the most common indicator of clinical efficacy in cancer and is an important indicator of person-centered clinical care [18]. Further detailed survival analysis of LGG subtypes confirmed the adverse impact on the prognosis of patients with Grade III LGG due to the relatively high expression in Grade III LGG. Moreover, the effect of *WDR76* on the poor prognosis in patients with LGG was not influenced by the well-known molecular characteristics, wild-type IDH or 1p19q non-co-deletion, which are recognized as key markers of more vicious LGG subtypes in the 2016 WHO classification criteria [19]. Thus, we believe that *WDR76* identified in this study can be juxtaposed with these two molecular features as markers for different subtypes of LGG. In addition, wild-type IDH and 1p19q non-co-deletion have been widely proven to significantly reduce patient survival [20], and the present study also confirmed that *WDR76* was an independent prognostic risk factor for LGG using the multivariate Cox regression analysis. These results further supported our hypothesis. In the 2021 WHO classification of tumors, additional molecular features have been introduced for glioma classification [21]. Therefore, in the near future, the high expression of *WDR76* observed in this study may also be incorporated into glioma diagnosis as a standard molecular characteristic for the malignant subtype of LGG with a poor prognosis.

In this study, the high expression of *WDR76*, which contributes to the progression of LGG malignancy, was confirmed to be regulated by DNA methylation. Gene methylation is an important component of epigenetic regulation and has been increasingly studied in gliomas. MGMTp methylation status, an authoritative molecular feature included in the 2016 WHO glioma subtype, represents the sensitivity of gliomas to chemotherapeutic agents and provides a reliable reference for the selection of clinical treatment options [22]. In addition, a recent study reported that the low methylation status of four CpG sites of podocan-like 1 (*PODNL1*) is significantly associated with poor OS and can be used to predict shorter disease-free survival [23]. Similar results were also confirmed in this study, which showed that methylation of the cg10739344 and cg18592307 sites, which inhibited the expression of *WDR76*, blocked the malignant progression of LGG and prolonged the OS of patients with LGG. In addition, both methylation sites of *WDR76* can be independently used as favorable prognostic factors. These results further enrich the understanding of regulatory mechanisms at the molecular level of *WDR76*, which can inhibit the undesirable progression of LGG to a higher malignancy by upregulating the methylation levels of key CpG sites and decreasing the expression of *WDR76*, thereby reducing downstream signaling pathway activation. Furthermore, the detailed regulatory mechanisms for determining each methylation site in *WDR76* require further investigation using third-generation sequencing.

This study elucidated the molecular level alterations and downstream pathway activations that are triggered by the abnormally high expression of *WDR76* in LGG to promote the progression of LGG malignancy. Co-expression analysis revealed that the five genes most positively associated with *WDR76* were involved in spindle assembly and chromosome segregation during mitosis, thus regulating the cell cycle progression [24–28]. Abnormally high expression of these genes, which are involved in cell proliferation, has also been shown to drive poor progression in multiple cancers. For example, aberrantly high expression of *PRC1*, the most positively correlated gene, is associated with a poor prognosis in patients with adrenocortical carcinoma. Another co-expressed gene, *NUSAP1*, is also recognized as an independent risk factor for HCC patient outcome, and regulates the expression of CDK4, CDK6, and cyclinD1, which participate in cell cycle progression [29]. Similar results have shown that ASF1B promotes aggressive features of invasion and metastasis in lung cancer cells via the p53 signaling pathway [30]. In addition, studies have confirmed that the positively correlated co-expression genes *NUSAP1*, *ASF1B*, *TPX2*, and *SKA1* are invariably involved in immune cell infiltration in numerous cancers, and thus contribute to the regulation of the tumor immune microenvironment [29, 31–33]. In summary, molecular changes induced by *WDR76* synergistically mediate the malignant progression of LGG, and genes co-expressed with similar functions revealed the tip of the iceberg that *WDR76* is involved in malignant mechanisms.

Furthermore, signaling pathway enrichment analysis confirmed the consistency of the GSEA results with the co-expression analysis showing that *WDR76* participates in cell cycle signaling pathways to promote the malignancy of LGG. *WDR76* is also involved in other oncogenic pathways, such as the Notch signaling pathway. The well-known Notch pathway dominates the malignant features of cancers, such as epithelial–mesenchymal transition and angiogenesis [34], and is involved in regulating the immune microenvironment to achieve tumor-induced immunoprecipitation [35]. Moreover, the p53 signaling pathway was also included in the enrichment results, in which mutant p53 lost its tumor-suppressive function [36]. In addition, the mismatch repair pathway in the GSEA results has been reported to antagonize DNA damage induced by radiochemotherapy in tumor cells and ultimately mediate therapeutic resistance in cancer cells [37]. Both p53 signaling and mismatch repair pathway have been demonstrated to be involved in regulating the cancer immune microenvironment [38, 39]. Taken together, this study details the signaling pathway through which *WDR76* participates in LGG pathology, demonstrates the potential key mechanisms of carcinogenicity of *WDR76*, and expands the understanding of etiology and immunotherapy for LGG.

With the popularity of immunotherapies in the LGG landscape, the role of *WDR76* in tumor immune mechanisms may be a breakthrough to further improve patient outcomes. For example, M2 macrophages are particularly prominent among the infiltrating immune cells promoted by *WDR76*. Alternatively, activated (M2) macrophages have been shown to promote glioma cell proliferation, elevate the expression of anti-inflammatory cytokines, and increase glioma cell invasion, ultimately leading to glioma progression and immune escape [40]. In this study, *WDR76* not only contributed to the infiltration of M0 macrophages but also preferred M2 polarization, thus creating an inhibitory immune microenvironment that is more favorable for LGG progression. At the molecular level, the positive association between *WDR76* and numerous immune checkpoints highlighted its importance in immunotherapy. Neoadjuvant administration of PD-1 blockade in current immunotherapy promotes T-cell cloning and the expression of relevant genes, further improving local and systemic anti-tumor immune responses [41]. In addition, *CD276* not only predicts the poor prognosis in glioma [42], but also serves as an effective target for chimeric antigen receptor T cells targeting glioma [43]. Therefore, *WDR76* may serve as a novel therapeutic target to improve the clinical efficacy of immunotherapy in combination with the existing therapies based on these immune checkpoints. Finally, as a target candidate for immunotherapy, *WDR76* will further enrich the options for LGG immunotherapies, deepen and strengthen the link between cellular and molecular therapies, and provide unprecedented opportunities for improving the comprehensive treatment of LGG.

## Conclusion

Taken together, this study is the first to confirm *WDR76* as a prognostic risk factor for lower grade glioma, which may influence the progression of malignancy through its involvement in the cell cycle and the Notch signaling pathway. Furthermore, *WDR76* mediates tumor immunity in lower grade glioma by regulating the infiltration of immune cells such as M2 macrophages into the immune microenvironment. This study not only expands knowledge on the molecular function of *WDR76*, but also provides a promising target molecule for future precision therapies and immunotherapy of lower grade glioma, offering insights to improve patient survival outcomes.

### Supplementary Information

**Additional file 1: Table S1.** Detailed clinical features of LGG patients in TCGA RNA-seq. **Table S2.** Detailed clinical features of LGG patients in CGGA RNA-seq. **Table S3.** Gene set enriches the high *WDR76* expression phenotype based on TCGA–RNA-seq data and CGGA–RNA-seq data. **Figure S1.** Prognostic value of *WDR76* in LGG with different WHO grades and molecular features. **Figure S2.** Prognostic value of methylation sites of *WDR76* in LGG. **Figure S3.** Relationship between immune cell infiltration and overall survival in LGG patients.

## Data Availability

Publicly available data sets were analyzed in this study. This data can be found here: https://www.cancer.gov/about-nci/organization/ccg/research/structural-genomics/tcga; http://www.cgga.org.cn/; https://www.ncbi.nlm.nih.gov/geo/.

## Abbreviations

WDR: The WD40 repeat
*WDR76*: WD repeat domain 76
LGG: Lower grade glioma
GBM: Glioblastoma
PPI: The protein–protein interaction
CRC: Colorectal cancer
DSS: Disease-specific survival
GEPIA: The gene expression profiling interactive analysis
TCGA: The cancer genome atlas
CGGA: The Chinese Glioma Genome Atlas
GEO: The gene expression omnibus database
TIMER: The tumor immune estimation resource database
GSEA: Gene set enrichment analysis
FDR: False discovery rate
NCBI: National center for biotechnology information
FBS: Fetal bovine serum
KM: Kaplan–meier
OS: Overall survival
ROC: Receiver operating characteristic
ESTIMATE: Estimation of STromal and Immune cells in MAlignant Tumour tissues using Expression data
AA: Anaplastic astrocytoma
rAO: Recurrent anaplastic oligodendroglioma
HCC: Hepatocellular carcinoma
CRC: Colorectal cancer
LUAD: Lung adenocarcinoma
DFS: Disease-free survival
ACC: Adrenocortical carcinoma
HCC: Hepatocellular carcinoma
EMT: Epithelial–mesenchymal transition
CAR-T: Chimeric antigen receptor T
*PODNL1*: Podocan-like 1
