# Supplementary material for: WD repeat domain 76 predicts poor prognosis in lower grade glioma and provides an original target for immunotherapy
Source: Eur J Med Res. 2024 Jan 3;29:13. doi: 10.1186/s40001-023-01605-6 (PMC10763342; doi:10.1186/s40001-023-01605-6)
Supplement: Supplementary file 1 — Additional file 1: Table S1. Detailed clinical features of LGG patients in TCGA RNA-seq. Table S2. Detailed clinical features of LGG patients in CGGA RNA-seq. Table S3. Gene set enriches the high WDR76 expression phenotype based on TCGA–RNA-seq data and CGGA–RNA-seq data. Figure S1. Prognostic value of WDR76 in LGG with different WHO grades and molecular features. Figure S2. Prognostic value of methylation sites of WDR76 in LGG. Figure S3. Relationship between immune cell infiltration and overall survival in LGG patients. [file 40001_2023_1605_MOESM1_ESM.docx]

**
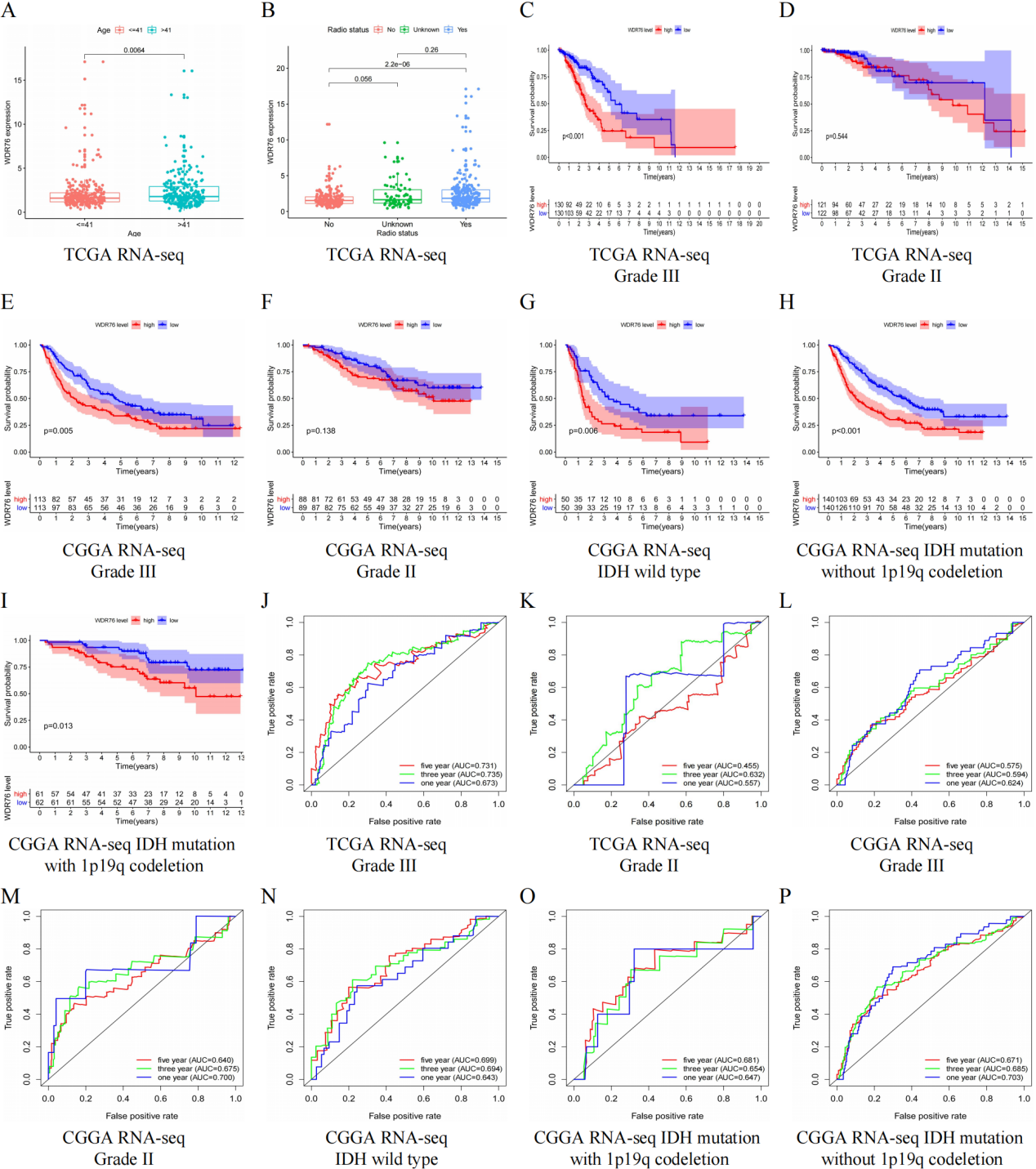
**

**Figure S1.** The prognostic value of *WDR76* in LGG with different WHO grades and molecular features. (**A-B**) Age and Radio status based on TCGA database. (**C-F**) Overall survival of patients with WHO grade II and III LGG in both databases. (**G-I**) Overall survival of LGG patients with different molecular features in CGGA database. (J**-M**) The ROC curves of WHO grade II and III LGG in both databases. (**N-P**) The ROC curves of LGG with different molecular features in CGGA database.

**
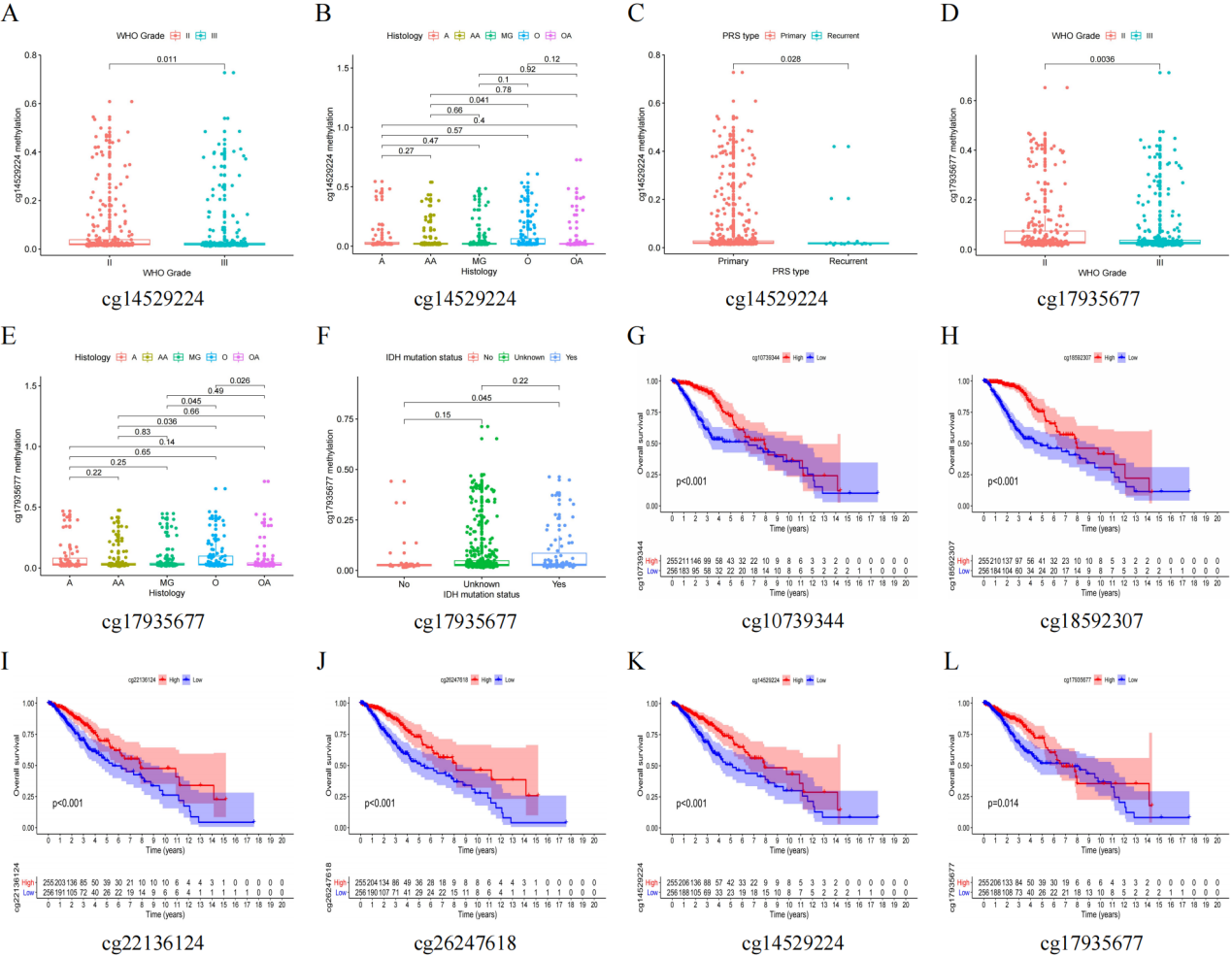
Figure S2.** The prognostic value of methylation sites of *WDR76* in LGG. (**A-C**) The relationship between methylation status of cg14529224 with WHO Grade, Histology, and PRS type. (**D-F**) The relationship between methylation status of cg17935677 with WHO Grade, Histology, and IDH mutation status. (**G-L**) The overall survival in LGG patients with high and low methylation levels of cg10739344, cg18592307, cg22136124, cg26247618, cg14529224, and cg17935677 in *WDR76*.


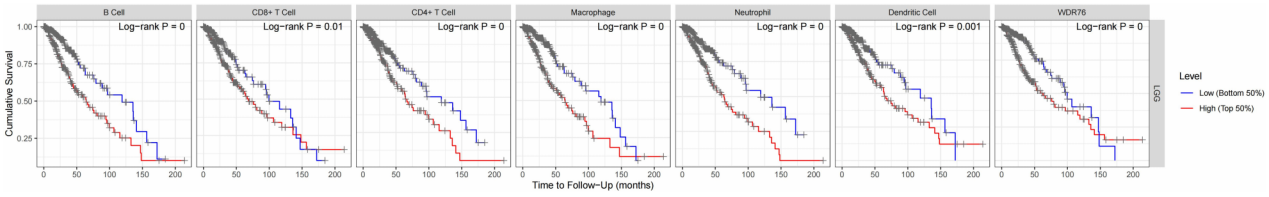


**Figure S3.** The relationship between immune cell infiltration and overall survival in LGG patients.

**Table S1 The detailed clinical features of LGG patients in TCGA RNA-seq**

| **Covariates** | **Type** | **Total** | **Percentages (%)** |
| --- | --- | --- | --- |
| Age | <=41 | 260 | 51.69% |
|  | >41 | 243 | 48.31% |
| Gender | Female | 225 | 44.73% |
|  | Male | 278 | 55.27% |
| WHO Grade | II | 243 | 48.31% |
|  | III | 260 | 51.69% |
| Radio status | No | 187 | 37.18% |
|  | Unknown | 72 | 14.31% |
|  | Yes | 244 | 48.51% |
| Chemo status | No | 167 | 33.20% |
|  | Unknown | 66 | 13.12% |
|  | Yes | 270 | 53.68% |
| PRS type | Primary | 489 | 97.22% |
|  | Recurrent | 14 | 2.78% |
| IDH mutation status | No | 34 | 6.76% |
|  | Unknown | 378 | 75.15% |
|  | Yes | 91 | 18.09% |
| expression | High | 251 | 49.90% |
|  | Low | 252 | 50.10% |
| methylation | High | 251 | 49.90% |
|  | Low | 252 | 50.10% |

**Table S2 The detailed clinical features of LGG patients in CGGA RNA-seq**

| **Covariates** | **Type** | **Total** | **Percentages (%)** |
| --- | --- | --- | --- |
| PRS type | Primary | 273 | 67.74% |
|  | Recurrent | 130 | 32.26% |
| WHO Grade | II | 177 | 43.92% |
|  | III | 226 | 56.08% |
| Gender | Female | 171 | 42.43% |
|  | Male | 232 | 57.57% |
| Age | <=41 | 222 | 55.09% |
|  | >41 | 181 | 44.91% |
| Radio status | No | 88 | 21.84% |
|  | Yes | 315 | 78.16% |
| Chemo status | No | 134 | 33.25% |
|  | Yes | 269 | 66.75% |
| IDH mutation | No | 100 | 24.81% |
|  | Yes | 303 | 75.19% |
| 1p19q codeletion | No | 280 | 69.48% |
|  | Yes | 123 | 30.52% |
| MGMTp methylation | No | 165 | 40.94% |
|  | Yes | 238 | 59.06% |

**Table S3. The gene set enriches the high WDR76 expression phenotype based on TCGA-RNA seq data and CGGA-RNA seq data.**

| **Gene set name** | **TCGA-RNA seq** | | | **CGGA-RNA seq** | | |
| --- | --- | --- | --- | --- | --- | --- |
|  | **NES** | **NOM p-val** | **FDR q-value** | **NES** | **NOM p-val** | **FDR q-value** |
| CELL_CYCLE | 2.2098007 | 0 | 8.01E-04 | 2.0979135 | 0 | 0.01586634 |
| NOTCH_SIGNALING_PATHWAY | 1.6627773 | 0.04106776 | 0.08820902 | 1.6895618 | 0.012096774 | 0.09834949 |
| P53_SIGNALING_PATHWAY | 2.1459174 | 0 | 0.001160608 | 1.7859939 | 0.006012024 | 0.09604696 |
| MISMATCH_REPAIR | 2.0740213 | 0 | 0.004387569 | 1.8839073 | 0 | 0.046782635 |

NES: normalized enrichment score; NOM: nominal. Gene sets with NOM P-value <0.05 and FDR q-value <0.25 were considered as significantly enriched.
